# Preparation and Photocatalytic Degradation Performance of C@Cd_x_Mn_1−x_S to Tetracycline Hydrochloride

**DOI:** 10.3390/ma18051062

**Published:** 2025-02-27

**Authors:** Yabin Su, Zedong Zeng, Haowen Chen, Zuosheng Lv, Cen Tan, Congjin Chen

**Affiliations:** Guangxi Key Laboratory of Petrochemical Resources Processing and Process Intensification Technology, School of Chemistry and Chemical Engineering, Guangxi University, Nanning 530004, China; 18934709339@163.com (Y.S.); 15586229137@163.com (Z.Z.); 19976192847@163.com (H.C.); sense1ess030503@163.com (Z.L.); tancen23@163.com (C.T.)

**Keywords:** biomass gasification carbon residue, C@Cd_x_Mn_1−x_S, composite catalyst, photocatalytic degradation, tetracycline hydrochloride

## Abstract

Cd_x_Mn_1−x_S solid solutions were synthesized by incorporating Mn^2+^ into CdS and the optimal ratio of Mn^2+^ to Cd^2+^ was explored via photocatalytic degradation performance for tetracycline (TC). Subsequently, the composite catalyst C@Cd_x_Mn_1−x_S was prepared by loading Cd_x_Mn_1−x_S onto the biomass gasification carbon residue (C) by hydrothermal method and characterized by various characterization tests. The optimal TC photodegradation condition and degradation mechanism catalyzed by C@Cd_x_Mn_1−x_S was investigated. The results showed Cd_0.6_Mn_0.4_S had the optimal photocatalytic degradation efficiency, which is about 1.3 times that of CdS. The TC photodegradation efficiency by C@Cd_0.6_Mn_0.4_S prepared at the mass ratio of C to Cd_0.6_Mn_0.4_S of 1:2 was the best, which was 1.24 times that of Cd_0.6_Mn_0.4_S and 1.61 times that of CdS. Under the optimal conditions (visible light irradiation for 60 min, C@Cd_0.6_Mn_0.4_S of 20 mg, 40 mL TC solution of 40 mg/L), the TC degradation efficiency was 90.35%. The degradation efficiencies of 20 mg/L levofloxacin, ciprofloxacin, and 40 mg/L oxytetracycline catalyzed by C@Cd_0.6_Mn_0.4_S range from 89.88% to 98.69%. In the photocatalytic reaction system, •O_2_^−^ and h^+^ are the dominant active species, which directly participate in the photocatalytic degradation reaction of TC, and •OH contributes little. The work provides a strategy to improve the photocatalytic performance of CdS for photocatalytic degradation antibiotics, and opens an interesting insight to deal with solid waste from biomass gasification.

## 1. Introduction

Among the organic pollutants existing in the aquatic environment, antibiotics have received special attention. Antibiotics are secondary metabolites or synthetic analogues produced by bacteria, mold, or other microorganisms. They are widely used in the treatment of infectious diseases in humans and animals, and are also widely used in aquaculture and livestock breeding. Antibiotics are not fully absorbed in humans and animals, and most of them enter waste treatment plants or enter the environment directly in the form of raw or active metabolites. Although the half-life of most antibiotics is short, due to their frequent use and entry into the environment, they have posed potential ecological risks to the ecological environment and human health [1,2]. Antibiotics and their metabolites have been detected in surface water, groundwater, sewage, and drinking water, with concentrations ranging from mg/L to μg/L [1,2]. Tetracycline is one of the antibiotics that widely exists in the aquatic environment [3]. However, tetracycline has a stable structure. Removal methods of tetracycline from water usually involve in biodegradation, adsorption, Fenton or Fenton-like reaction, persulfate advanced oxidation process, photocatalytic oxidative degradation, etc. Among these methods, biodegradation requires specific microbes and takes a long time, adsorption may cause secondary pollution, and Fenton or Fenton-like reaction and persulfate advanced oxidation process all require additional chemicals. Therefore, the green, harmless, and simple-to-operate semiconductor photocatalytic technology is often used to degrade tetracycline [3,4,5].

Many semiconductor photocatalysts can perform efficient photocatalysis reaction under visible light, such as TiO_2_, ZnO, Bi_2_WO_6_, Bi_2_MoO_6_, g-C_3_N_4_, CdS, etc. [6,7,8,9]. However, most semiconductors have shortcomings such as high price, wide band gap, and low efficiency [10]. Among existing photocatalysts, CdS is a metal semiconductor with a narrow band gap (2.4 eV). However, the photogenerated charge carrier recombination rate of CdS alone is high, there is the photocorrosion, and CdS is prone to agglomeration, resulting in low efficiency, which greatly restricts its practical application [7]. From the existing reports, it can be summarized that the regulation of physics factors such as morphology, crystal structure, crystallinity, and particle size have a significant impact on the performance of CdS [11]. CdS can be prepared into nanorods [12], nanosheets [13], nanotubes [14], hollow structures [15], etc. Constructing a heterojunction by combining CdS catalysts with other materials [8,9,16,17] or loading CdS catalysts on a carrier with a large specific surface area [18,19] are common methods to solve the problem of CdS photocorrosion. Among carriers with a large specific surface area, the biomass gasification carbon residue (C) contains some hydrophilic/lipophilic functional groups and aromatic carbon structures, as well as a high carbon content and developed porous structure. It has been reported to be used for the catalyst’s carrier [20]. It is speculated that the biomass gasification carbon residues can be used as the loading material of CdS-based photocatalysts to increase the contact chance between photocatalysts and contaminants, and increases the exposure of the active sites of photocatalysts, realizing the effective separation of photo-induced carriers, hindering the rapid recombination of photo-induced carriers, and thereby improving the photocatalytic activity.

Solid solution strategy is also a feasible method to modify CdS catalysts [21]. A solid solution is performed by selecting another metallic element of atom size and properties similar to the Cd atom for ion exchange at a specific location in the crystal structure without changing the overall crystal structure or symmetry. The photocatalytic activity and stability of solid solution materials can be improved by adjusting the ratio of metal ions to change the band position and band gap width. Researchers often construct solid solutions with CdS by introducing transition metals such as Zn, Mn, and Cu [22,23,24,25,26,27,28]. Wang et al. [29] prepared a series of 3D dendritic Mn_x_Cd_1−x_S (LMCS-x) photocatalysts using a simple hydrothermal method without the addition of template agent and surfactant. With Pt as the co-catalyst, the photocatalytic cracking performance of aquatic hydrogen was tested. The hydrogen production efficiency of LMCS-2 is three times that of CdS.

Aimed to make up for the limitations of the single-component CdS and exert the synergy effect of CdS and MnS in Cd_x_Mn_1−x_S and thereby improve the photocatalytic performance and stability of the CdS photocatalyst, in the work, Cd_x_Mn_1−x_S solid solutions were synthesized by incorporating Mn^2+^ into CdS using a hydrothermal method. The optimal ratio of Mn^2+^ to Cd^2+^ was explored through various characterization tests and photocatalytic degradation performance evaluation. Subsequently, the composite catalyst C@Cd_x_Mn_1−x_S was prepared by loading Cd_x_Mn_1−x_S onto the biomass gasification carbon residue (C) by hydrothermal method, characterized by scanning electron microscope–Energy-dispersive X-ray spectroscopy (SEM-EDS), Fourier transform infrared spectroscopy (FTIR), X-ray diffraction (XRD), N_2_ adsorption–desorption isotherm, X-ray photoelectron spectroscopy (XPS), and other characterization methods. Taking TC degradation efficiency as the main index, the catalytic activity of C@Cd_x_Mn_1−x_S under different pH values, catalyst dosages, different TC concentrations, and reaction systems was explored; the reusability, stability, and universality of the composite catalyst were determined; finally, the mechanism of photocatalysis was inferred. The work will provide a strategy to improve the photocatalytic performance of CdS for photocatalytic degradation antibiotics, and opens an interesting insight to deal with solid waste from biomass gasification.

## 2. Materials and Methods

### 2.1. Reagents and Instruments

Phosphoric acid (H_3_PO_4_), thioacetamide CH_3_CSNH_2_ (TAA), ethanol (EtOH), sodium chloride (NaCl), sodium nitrate (NaNO_3_), sodium sulfate (Na_2_SO_4_), magnesium chloride (MgCl_2_), and hydrochloric acid (HCl) were purchased from Guangdong Guanghua Technology Co., Ltd. (Guangzhou, China). Cadmium nitrate tetrahydrate (Cd(NO_3_)_2_·4H_2_O), manganese acetate tetrahydrate (Mn(OAc)_2_·4H_2_O), humic acid (HA), ciprofloxacin (CIP), levofloxacin (LVFX), and oxytetracycline (OTC) were purchased from Shanghai Mclean Biochemical Technology Co., Ltd. (Shanghai, China) *p*-benzoquin (p-BQ) and tetracycline hydrochloride (TC) were purchased from Arald Inc. (Shanghai, China) Co., Ltd. Sodium hydroxide (NaOH), isopropyl alcohol (IPA), disodium EDTA (EDTA-2Na), sodium bicarbonate (NaHCO_3_), disodium hydrogen phosphate dehydrates (Na_2_HPO_4_·2H_2_O), and sodium carbonate (Na_2_CO_3_) were purchased from Sinopharm Group Chemical Reagent Co., Ltd., (Beijing, China). All reagents are analytical purity level. Biomass gasification carbon residues were provided by the Demonstration Project of Multi-Product Comprehensive Utilization Technology of Biomass Gasification. The water used in the experiment is deionized water.

The following instruments were used in this study: MiniFlex600 X-ray diffractometer (XRD), Rigaku, Tokyo, Japan; Niolet iN10 Fourier Transform Infrared Spectroscopy (FT-IR) microscope, K-Alpha X-ray photoelectron spectrometer (XPS), Thermo Fisher Scientific, Waltham, MA, USA; Zeiss Gemini 300 Field Emission Scanning Electron Microscope (SEM), Carl Zeiss, Jena, Germany; ASAP2460 Fully Automatic BET Surface Area and Porosity Analyzer, Micromeritics, Norcross, GA, USA; FLS1000 Steady-state/Transient Fluorescence Spectrometer (PL), Edinburgh, UK; CHI 650E Electrochemical Workstation, Shanghai Chenhua Instrument Co., Ltd., Shanghai, China; DHG-9070 Electric Heating Constant Temperature Blower Drying Oven, Shanghai Jinghong Laboratory Equipment Co., Ltd., Shanghai, China; UV-2802 Ultraviolet-Visible Spectrophotometer, Shanghai UNIC Instruments Co., Ltd., Shanghai, China. 

### 2.2. Preparation Method

#### 2.2.1. Preparation of CdS and MnS

CdS (MnS) nanomaterials were synthesized by hydrothermal methods. 2 mmol of Cd (NO_3_)_2_·4H_2_O (Mn (OAc)_2_·4H_2_O) was dissolved in 20 mL of deionized water. After complete dissolution, 2.5 mmol of TAA was added and dissolved, followed by the addition of 10 mL of 2M NaOH. After continuous stirring for 30 min, the mixture was put into an autoclave and reacted at 180 °C for 12 h. After cooling, the products were filtered, washed alternately with deionized water and ethanol several times and dried for 6 h at 70 °C under vacuum; CdS (MnS) was obtained, then ground and stored for later use.

#### 2.2.2. Preparation of Cd_x_Mn_1−x_S

The Cd_x_Mn_1−x_S solid solution was synthesized by hydrothermal methods. An appropriate amount of Mn (OAc)_2_·4H_2_O and Cd (NO_3_)_2_·4H_2_O were dissolved in 20 mL of deionized water. The molar ratios of Mn^2+^ to Cd^2+^ were controlled as 5:0, 4:1, 3:2, 2:3, 1:4, and 0:5, respectively (Cd^2+^ + Mn^2+^ = 2 mmol). After complete dissolution, 2.5 mmol TAA was added and dissolved, followed by the addition of 10 mL of 2 mol/L NaOH. After continuous stirring for 30 min, the mixture was put into an autoclave and reacted at 180 °C for 12 h. After cooling, the sample was filtered, washed alternately with deionized water and ethanol several times, and dried for 6 h at 70 °C under vacuum; Cd_x_Mn_1−x_S was obtained and labeled as Cd_x_Mn_1−x_S (x = 0, 0.2, 0.4, 0.6, 0.8, 1). Then each sample was ground and stored for later use.

#### 2.2.3. Treatment of Biomass Gasification Carbon Residue

Firstly, the original biomass gasification residues were ground through a 100-mesh sieve; 10.000 g of the sieved biomass gasification residue was poured into a 200 mL NaOH (1 mol/L) aqueous solution and stirred for 12 h at 80 °C to remove silicates. After filtration and washing, the washed biomass gasification residue was poured into a 200 mL H_3_PO_4_ (1 mol/L) aqueous solution and stirred for 12 h at room temperature to remove inorganic metal ions. Then, the carbon powder after acid washing was washed alternately with deionized water and absolute ethanol until neutral, suction filtered, dried at 70 °C for 12 h; the sample was then ground to obtain the biochar (C), and preserved for the subsequent use.

#### 2.2.4. Preparation of C@Cd_0.6_Mn_0.4_S Composite Catalyst

**Scheme 1:**
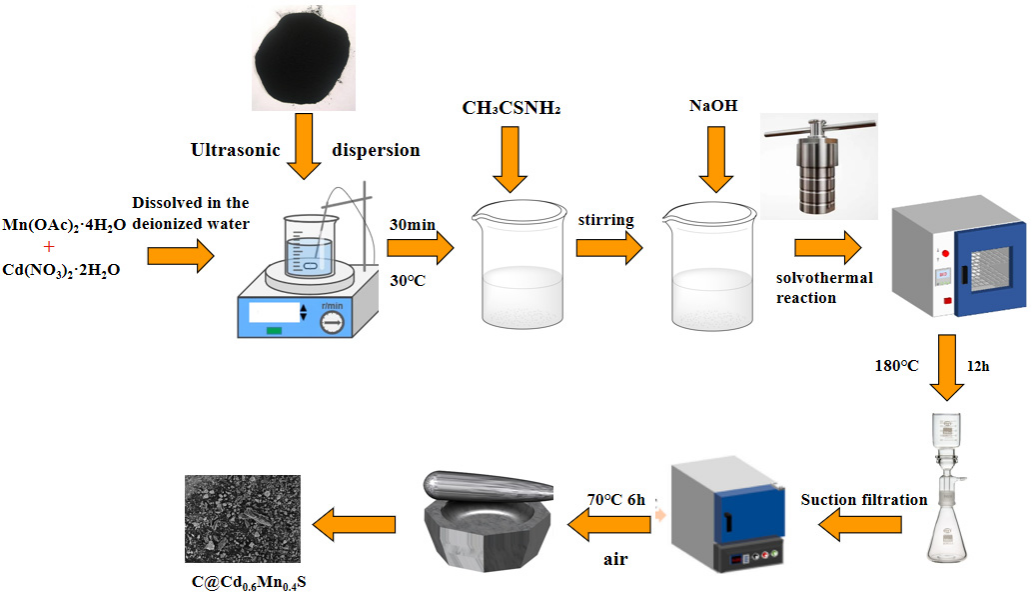
The preparation process of C@Cd_x_Mn_1−x_S.

The C@Cd_0.6_Mn_0.4_S composite materials with different mass ratios of C to C@Cd_0.6_Mn_0.4_S (labeled as C@Cd_0.6_Mn_0.4_S (1:X), where X = 1, 2, 3, 4) were obtained by introducing biochar (C) as an auxiliary catalyst by chemical deposition. Quantities of 0.8 mmol Mn (OAc)_2_·4H_2_O and 1.2 mmol Cd (NO_3_)_2_·4H_2_O were put into 20 mL deionized water and completely dissolved. Then, C (C: Cd_0.6_Mn_0.4_S with mass ratio of 1:1, 1:2, 1:3, and 1:4) was added. After ultrasonic treatment for 30 min, 2.5 mmol TAA was added, dissolved, and then 10 mL of 2 mol/L NaOH was added. After continuous stirring for 30 min, the mixture was put into an autoclave and reacted at 180 °C for 12 h. After cooling, the products were filtered, washed alternately with deionized water and ethanol several times, and dried for 6 h at 70 °C under vacuum. The preparation process of C@Cd_x_Mn_1−x_S is shown in Scheme 1.

### 2.3. Structural Characterization and Performance Testing

XRD test: the crystal form of the sample was detected by X-ray diffractometer at room temperature; Cu target; accelerating voltage: 40 kV; tube current: 40 mA; scanning range: 10° to 80°; scanning rate: 5 °/min. FTIR test: potassium bromide pellet method; scanning wavenumber range: 4000 to 400 cm^−1^; scanning times: 32 times, resolution: 4 cm^−1^. N_2_ adsorption–desorption test: N_2_ adsorption–desorption isotherms of the sample were obtained using an automatic specific surface and porosity analyzer; the degassing temperature and degassing time were 200 °C and 8 h, respectively. SEM test: accelerating voltage: 3.0 kV; gold-plating treatment. XPS test: Al Kα excitation source; energy: 1486.6 eV; the spectrometer calibration and charge correction were performed at the binding energy of C 1s of 284.8 eV. Photoluminescence (PL) test: utilized a steady-state/transient fluorescence spectrometer, with xenon lamp as the excitation light source; excitation wavelength: 380 nm; scanning range: 400–800 nm. Ultraviolet-visible diffuse reflectance (UV-Vis DRS, UV3600, Tokyo, Japan) test: with BaSO_4_ as the reference material, the measurement range is 200–800 nm.

### 2.4. Photodegradation of TC Catalyzed by C@Cd_0.6_Mn_0.4_S

A certain amount of C@Cd_0.6_Mn_0.4_S was poured into the photocatalytic test tube containing 40 mL of TC standard solution with a mass concentration of 40 mg/L. The mixture was stirred for 30 min under dark conditions to achieve adsorption–desorption equilibrium. Then, a photodegradation experiment was carried out in the photocatalytic reaction system equipped with a 300 W xenon lamp with a 420 nm cut-off filter. Then, 2 mL of the reaction solution was taken out and filtered through a 0.22 μm water system filter membrane at 15-min intervals; the absorbance (*A*) of filtrate of the reaction solution was determined at the maximum absorption wavelength (357 nm) of TC using an ultraviolet–visible spectrophotometer, and the mass concentration (*ρ*) of TC was calculated by the calibration curve Equation (1) for the relationship between the absorbance (*A*) and the mass concentration (*ρ*) of the TC standard solution. The degradation efficiency (*η*) of TC is calculated according to Equation (2).

*A* = 0.03159 *ρ* + 0.0057 (*R*^2^ = 0.9988) (1)

*η =* (1 − *ρ_t_/ρ*_0_) × 100% (2)

where *η* is the degradation efficiency of TC, %; *ρ_t_* is the mass concentration of TC at the degradation time *t*, mg/L; and *ρ*_0_ is the initial mass concentration of TC, mg/L.

The effects of different proportions of the composite catalyst, the dosage of the catalyst, different reaction systems, TC concentration, pH value, different anions (HPO_4_^2–^, HCO_3_^−^, NO_3_^−^, Cl^−^, CO_3_^2−^, SO_4_^2−^) on photocatalytic degradation efficiency of TC were investigated, respectively. Experiments on the stability and reusability of the catalyst were performed as follows: 20 mg of C@Cd_0.6_Mn_0.4_S was put into 40 mL of TC aqueous solution with a mass concentration of 40 mg/L at 30 °C to degrade TC. The time gradients were set as 15, 30, 45, and 60 min. Samples were taken out and filtered after each time gradient, and the absorbance of the filtrate was determined using a 2802-PC UV-visible spectrophotometer, Unico (Shanghai) Instrument Co., Ltd., Shanghai, China. The concentration of TC in the degraded solution was calculated using the calibration curve equation, and the degradation efficiency was calculated according to Formula (2). After the degradation experiment, C@Cd_0.6_Mn_0.4_S was collected using a magnet after catalytic reaction, washed repeatedly with deionized water and ethanol-water, and then filtered. It was placed in an oven and dried for 12 h at 70 °C for reuse. The catalyst was reused 5 times.

## 3. Results and Discussion

### 3.1. Characterization Results

#### 3.1.1. SEM and EDS Analysis

**Figure 1:**
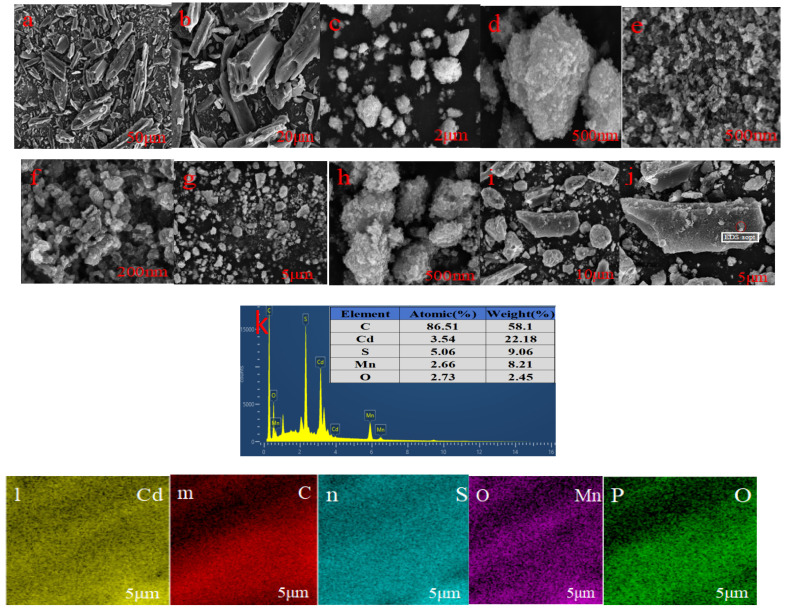
SEM images of C (**a**,**b**), CdS (**c**,**d**), MnS (**e**,**f**)**,** Cd 0.6Mn 0.4S (**g**,**h**), C@Cd_0.6_Mn_0.4_S (**i**,**j**), (**e**) EDS spectrum (**k**) and EDS elemental distribution map (**l**–**p**) of C@Cd_0.6_Mn_0.4_S.

Figure 1 shows the SEM images of biochar (a,b), CdS (c,d), MnS (e,f), C@Cd_0.6_Mn_0.4_S (g,h), and C@Cd_0.6_Mn_0.4_S (i,j), as well as the EDS images (k–p) of C@Cd_0.6_Mn_0.4_S (mass ratio of C:Cd_0.6_Mn_0.4_S = 1:2). Figure 1a,b indicate that biochar (C) has a fractured or intact columnar pore structure (the enlarged pores are clearly visible), which can load Cd_0.6_Mn_0.4_S. Figure 1c–f shows that CdS and MnS are aggregated from multiple spherical nanoparticles. As presented in Figure 1g,h, the as-prepared composite material C@Cd_0.6_Mn_0.4_S presents irregular nanoparticles and uneven distribution under different magnification levels, and these nanoparticles aggregate with each other, with an average particle size of less than 200 nm. As shown in Figure 1i,j, C@Cd_0.6_Mn_0.4_S nanoparticles grow closely on the surface of C, and the C surface, originally relatively smooth, becomes rough and the aggregation phenomenon of C@Cd_0.6_Mn_0.4_S weakens, which means that C@Cd_0.6_Mn_0.4_S can provide more active sites. As for the ratio of four elements of C, Cd, S, and Mn, Figure 1k shows that the atom percentage is 86.51%, 3.54%, 5.06%, and 2.66%, and weight percentage is 58.1%, 22.18%, 9.06%, and 8.21%, respectively. The atom ratio of Cd/Mn/S is about 0.70:0.53:1, not 0.6:0.4:1, which resulted for the following reasons: SEM–EDS testing is a powerful material surface analysis technique that can obtain information and chemical composition on the surface of a sample. However, the quantification of the chemical composition via SEM–EDS is a semi-quantitative method and is not accurate enough. In addition, O is originated from biochar. Figure 1l–p show that the elements of Cd, C, S, Mn, and O are uniformly distributed on the surface of the composite material, which also more fully demonstrates the growth and dispersion of Cd_0.6_Mn_0.4_S nanoparticles on the surface of C.

#### 3.1.2. XRD Analysis

**Figure 2:**
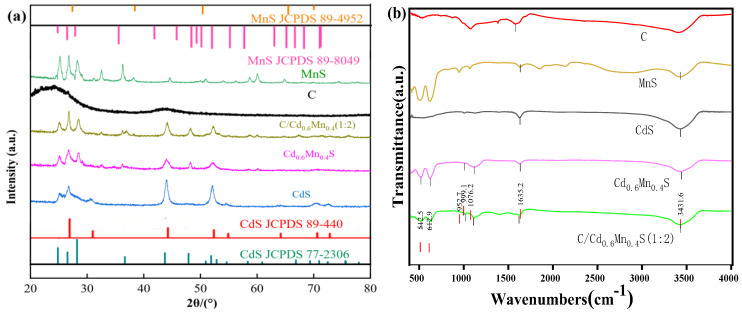
XRD patterns (**a**) and FTIR spectra (**b**) of C, CdS, MnS, Cd_0.6_Mn_0.4_S, and C@ C@Cd_0.6_Mn_0.4_S.

Figure 2a,b are the XRD patterns and FITR spectra of C, CdS, MnS, Cd_0.6_Mn_0.4_S, and C@Cd_0.6_Mn_0.4_S, respectively. In Figure 2a, biochar (C) presents a typical broad peak of amorphous carbon at 2θ = 26.6° ((002) reflection) and 45.3° ((100) reflection), indicating the formation of amorphous carbon [20,30]; MnS shows obvious diffraction peaks at 2θ = 25.8°, 27.7°, 29.3°, 38.2°, 45.5°, 53.0°, 54.1°, 55.0°, and 72.3°, which belong to the (100), (002), (101), (102), (110), (200), (112), (201), and (203) crystal faces of hexagonal γ-MnS (JCPDS No. 89-4089) [31,32], and 2θ = 29.3°, 35°, 62.1° and 75° belong to the (111), (200), (222), (200), (400) crystal faces of cubic α-MnS (JCPDS No. 89-4952) [32,33], indicating the obtained MnS is a hybrid of hexagonal γ-MnS and cubic α-MnS; the as-prepared CdS shows characteristic diffraction peaks: the peaks at 2θ = 24.9°, 26.5°, 44.0°, 52.1°, 70.0°, and 72.6° correspond to the (100), (002), (101), (112), (210), and (114) crystal faces, which are consistent with the standard peaks of hexagonal CdS (JCPDS 77-2306) [32]; the peaks at 2θ = 26.9, 31.0, 44.3, and 52.3° correspond to the (111), (200), (220), and (311) crystal faces of cubic CdS (JCPDS 89-440) [32], indicating the successful preparation of CdS and that the as-synthesized CdS is hybrid of hexagonal CdS and cubic CdS. By comparing the JCPDS standard cards of MnS and CdS, the characteristic peaks of the as-synthesized Cd_0.6_Mn_0.4_S solid solution are not a simple superposition of the peak shapes of CdS and MnS, indicating that the composite material is not a simple physical mixture of CdS and MnS, but that a special solid solution structure has been formed. This indicates that the Cd_0.6_Mn_0.4_S solid solution has been successfully formed. The diffraction peaks of C@Cd_0.6_Mn_0.4_S match those of Cd_0.6_Mn_0.4_S, and the XRD patterns of the two are almost the same. The as-prepared Cd_0.6_Mn_0.4_S and C@Cd_0.6_Mn_0.4_S are both hexagonal and cubic mixed crystal phase structures. In addition, XRD of C@Cd_0.6_Mn_0.4_S, since biochar is amorphous carbon, only has a broad peak response to the weak graphitized microcrystalline structure, and cannot be clearly observed in the composite material.

Figure 2b presents that the stretching vibration of –OH is at 3431.6 cm^−1^ in all samples; the stretching vibration of C=C/C=O bond is around 1635.2 cm^−1^ [34]; the vibration of C–O/C–C and C–H are overlapped at 1076.2 cm^−1^ [30]; the two peaks at 612.9 and 952.7 cm^−1^ are assigned the stretching vibration of Mn–S bond; and the stretching vibration of Cd–S bond is at 999.1 cm^−1^ [25,26,31,32]. However, the peaks of Mn–S bond and Cd–S bond in C@Cd_0.6_Mn_0.4_S are lower than those in pure MnS, CdS, and the Cd_0.6_Mn_0.4_S composite material_._ There are typical functional groups corresponding to C, as well as the characteristic peaks of CdS and MnS in the composite material, indicating that Cd_0.6_Mn_0.4_S is successfully loaded on biochar.

#### 3.1.3. XPS Analysis

**Figure 3:**
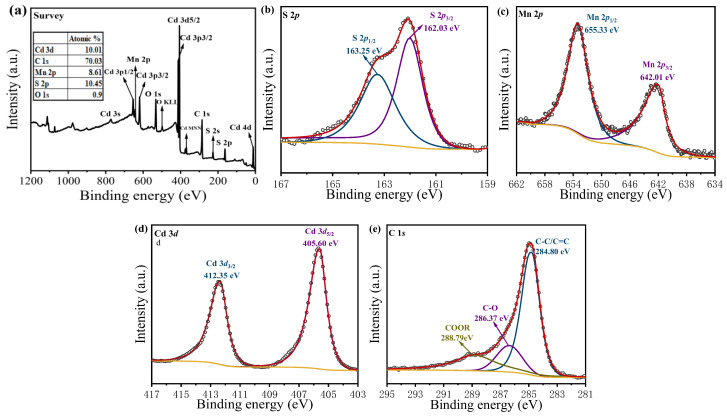
XPS spectra of C@Cd_0.6_Mn_0.4_S: (**a**) Survey spectrum; (**b**) S2p sub-spectrum; (**c**) Mn 2p sub-spectrum; (**d**) Cd 3p sub-spectrum; (**e**) C 1s sub-spectrum.

The composition and elemental valence state of the C@Cd_0.6_Mn_0.4_S solid solution was investigated by XPS. Figure 3a, the survey XPS spectra of the C@Cd_0.6_Mn_0.4_S solid solution, shows that the C@Cd_0.6_Mn_0.4_S is mainly composed of elements: Cd, Mn, S, C, and O; their atomic percentages are 10.01%, 8.61%,10.45%, 70.03%, and 0.9%, respectively. Among them, O is originated from biochar (the treated biomass gasification carbon residue). The results of element composition and content were almost consistent with those of SEM–EDS. Narrow-spectrum scanning of these elements was conducted to determine their valence states. As presented in Figure 3b, the S 2p spectrum can be divided into peaks at the binding energies of 163.25 and 162.03 eV, which matches well with S 2p_1/2_ and S 2p_3/2_ of S^2–^ in the solid solution [26]. As shown in Figure 3c, the two strong peaks at the binding energies of 655.33 and 642.01 eV correspond to Mn 2p_1/2_ and Mn 2p_3/2_, respectively, indicating that the Mn element exists in the catalyst in the form of Mn^2+^ [31,32]. As can be seen from Figure 3d, the two peaks at the binding energies of 405.60 and 412.35 eV correspond to Cd 3d_5/2_ and Cd 3d_3/2_, respectively, confirming that Cd exists in the catalyst in the form of Cd^2+^ [26]. In Figure 3e, the XPS spectrum of C 1s has a total of three peaks, corresponding to C=C/C–C (284.80 eV), C–O (286.37 eV), and C=O (288.79 eV) [35]. The peak of the C=C bond proves that C has a highly aromatic structure. The chemical valence states of Cd, Mn, and S in the solid solution existing in the form of Cd^2+^, Mn^2+^, and S^2−^ illustrated that Mn^2+^ has been successfully incorporated into the crystal structure of CdS, verifying the successful synthesis of the C@Cd_0.6_Mn_0.4_S solid solution.

#### 3.1.4. N_2_ Adsorption–Desorption Isotherm Analysis

**Figure 4:**
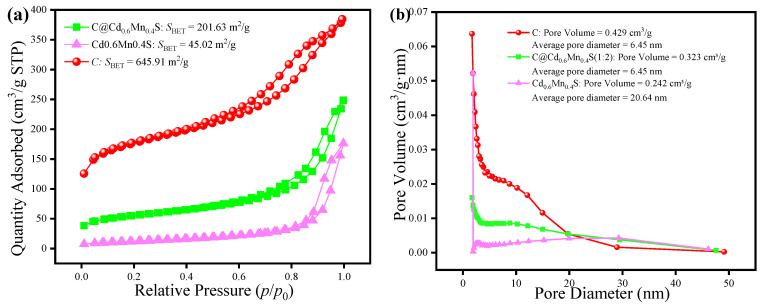
N_2_ adsorption–desorption isotherms (**a**) and pore size distribution diagrams (**b**) of C, C@Cd_0.6_Mn_0.4_S and Cd_0.6_Mn_0.4_S.

Figure 4a shows that the N_2_ adsorption–desorption isotherms of the three samples all belong to type IV according to the International Union of Pure and Applied Chemistry (IUPAC) classification of physisorption isotherms [36]. In the low-pressure region, the adsorption capacity slowly increases with the increase of pressure. Subsequently, with the increase of pressure at higher relative pressures, the adsorption amounts of the adsorption materials C (0.6–1), Cd_0.6_Mn_0.4_S (0.9–1.0), and C@Cd_0.6_Mn_0.4_S (0.8–1) increase suddenly. The desorption curves show obvious hysteresis loops, and the shape of the hysteresis loops belongs to type H3, indicating that the material has a slit pore structure caused by the aggregation of mesoporous particles and the stacking of flaky particles [36]. The specific surface area of the sample was calculated by Brunauer–Emmett–Teller (BET) methods, and the pore volume and pore size of the sample were calculated by the Barrett–Joyner–Halenda (BJH) methods. Figure 4b shows that the pore diameters of the three materials are unevenly distributed within the range of 1–49 nm, mainly concentrated in the range of 1–12 nm, which further proves that the material presents a mesoporous structure. Biochar exhibits a relatively high specific surface area (645.91 m^2^/g), and the specific surface area of C@Cd_0.6_Mn_0.4_S (201.63 m^2^/g) is larger than that of sole Cd_0.6_Mn_0.4_S (45.02 m^2^/g), indicating that Cd_0.6_Mn_0.4_S are loaded on the surface of biochar pores. The loaded Cd_0.6_Mn_0.4_S can significantly improve the dispersibility of Cd_0.6_Mn_0.4_S nanoparticles to expose more reaction sites for the catalyst and enhance the photocatalytic activity of the material.

#### 3.1.5. The Optical Absorption Properties and Energy Band Potential Analysis

**Figure 5:**
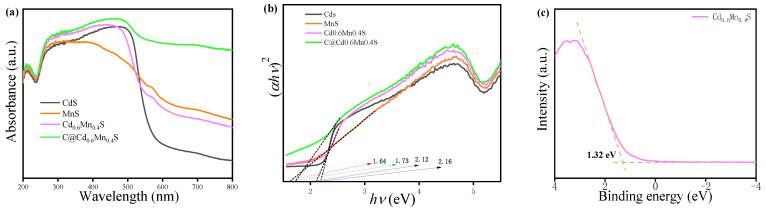
UV–Vis DRS absorbance spectra (**a**), (αh*v*)^2^ vs. h*v* plots (**b**) of CdS, Cd_0.6_Mn_0.4_S, C@Cd_0.6_Mn_0.4_S and MnS, and XPS valence band spectrum of Cd_0.6_Mn_0.4_S (**c**).

The UV–Vis light absorption performance of CdS, MnS, Cd_0.6_Mn_0.4_S, and C@Cd_0.6_Mn_0.4_S was investigated via UV–Vis diffuse reflectance absorption spectroscopy (UV-Vis DRS). As shown in Figure 5a, compared with CdS, the visible light absorption of Cd_0.6_Mn_0.4_S has a significant improvement. It can be speculated that the introduction of Mn^2+^ and biochar leads to an enhancement for its visible light absorption. The energy bandgap (*E_g_*) of the materials is estimated by the Kubelta–Munk equation (αh*v*)^2^ = A(h*v* − *E_g_*), where α, *v*, h, and A are the absorption coefficient, light frequency, Planck’s constant, and a constant, respectively [26,37], and is shown in Figure 5b. *E_g_* of CdS, MnS, Cd_0.6_Mn_0.4_S, and C@Cd_0.6_Mn_0.4_S are 2.16, 1.64, 2.12, and 1.73 eV, respectively. Due to the introduction of Mn^2+^ and biochar, *E_g_* of C@Cd_0.6_Mn_0.4_S decreases and is less than that of Cd_0.6_Mn_0.4_S and CdS. Based on the formula *λ* = 1240/*E_g_* [37] (*E_g_* is the energy bandgap, eV; *λ* is the excitation wavelength, nm), *λ* increases as *E_g_* decreases, which causes an absorption wavelength of C@Cd_0.6_Mn_0.4_S redshift, and enables C@Cd_0.6_Mn_0.4_S to have a higher visible light absorption ability. Based on the test data of valence band X-ray photoelectron spectroscopy (VB XPS), the linear part of the obtained graph near 0 eV is extrapolated to intersect with the horizontal extension line, the intersection point is the valence band potential (*E_VB_*), and *E_VB_* of Cd_0.6_Mn_0.4_S as determined by an XPS valence band analysis is 1.32 eV (Figure 5c, measured from the Fermi level)_._ According to the empirical formula *E_CB_* = *E_VB_ − E_g_*, the conduction band potential (*E_CB_*) of Cd_0.6_Mn_0.4_S is calculated as –0.80 eV.

### 3.2. Selection of C@Cd_0.6_Mn_0.4_S and Its Photocatalytic Degradation Performance

#### 3.2.1. Selection of Cd_0.6_Mn_0.4_S

**Figure 6:**
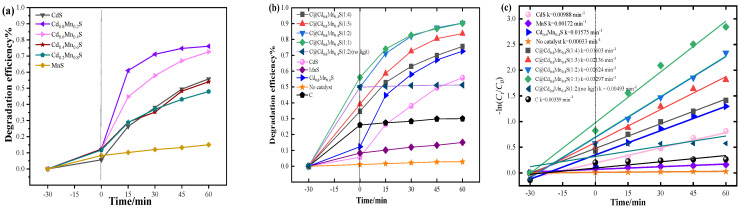
Effects of Cd_x_Mn_1−x_S with different proportions on the degradation efficiency of TC (**a**); the influence of catalysts at different mass ratio of biochar to Cd_0.6_Mn_0.4_S on the degradation efficiency of TC (**b**); and the quasi-first-order kinetics fitting (**c**) (Reaction conditions: the dosage of each catalyst was 20 mg, the mass concentration of TC solution was 40 mg/L, the solution volume was 40 mL, and the temperature was 30 °C).

The photocatalytic degradation of tetracycline hydrochloride (TC) was carried out, catalyzed by the solid solutions Cd_x_Mn_1−x_S (x = 0, 0.2, 0.4, 0.6, 0.8, 1). Firstly, each solid solution of Cd_x_Mn_1−x_S (x = 0, 0.2, 0.4, 0.6, 0.8, 1) with a mass of 20 mg was put into 40 mL of TC aqueous solution (40 mg/L). After holding for 30 min under non-illumination conditions to reach the adsorption–desorption equilibrium, the reaction lasted for 60 min under visible light conditions, and the absorbance was measured every 15 min. The effects of different solid solutions of Cd_x_Mn_1−x_S (x = 0, 0.2, 0.4, 0.6, 0.8, 1) on the degradation efficiency of TC were different. The results are shown in Figure 6a. When the amount of Mn^2+^ was doped with an appropriate ratio of Mn^2+^ to Cd^2+^, the photocatalytic activity of the synthesized solid solution increased significantly. However, when the amount of the doped Mn^2+^ was too high, the photocatalytic activity decreased instead. When x = 1, Cd_x_Mn_1−x_S is CdS, and the catalytic activity of CdS decreased within the photoreaction process due to its susceptibility to photocorrosion. Cd_0.8_Mn_0.2_S and Cd_0.6_Mn_0.4_S were obtained after doping Mn^2+^ to CdS; their stability was significantly improved compared to CdS, and the catalytic effect was also significantly enhanced. However, because the absorbance of MnS to light is lower than that of CdS, with the increase in the doping amount of Mn^2+^, the photosensitivity of Cd_x_Mn_1−x_S significantly decreases, resulting in a reduction in photocatalytic activity. Therefore, considering the activity comprehensively, stability, and the degradation efficacy of the Cd_x_Mn_1−x_S photocatalysis, the degradation efficacy of Cd_0.8_Mn_0.2_S and Cd_0.6_Mn_0.4_S does not differ much with the extension of degradation time, and Cd_0.8_Mn_0.2_S has less Mn^2+^ doped, which cannot clearly explore the influence of Mn^2+^ doping on CdS. Therefore, Cd_0.6_Mn_0.4_S was selected as the optimized material for photocatalytic degradation of TC. The photodegradation efficiency of TC catalyzed by Cd_0.6_Mn_0.4_S was about 1.3 times that catalyzed by CdS.

#### 3.2.2. Selection of the Mass Ratio of Biochar to Cd_0.6_Mn_0.4_S in C@Cd_0.6_Mn_0.4_S

After combining biochar with Cd_0.6_Mn_0.4_S at different mass ratios (during the preparation, the mass ratio of biochar to Cd_0.6_Mn_0.4_S = 1:X, X = 1, 2, 3, 4), Cd_0.6_Mn_0.4_S was loaded on biochar. The photodegradation efficiency of TC by catalysts at different mass ratios of biochar to Cd_0.6_Mn_0.4_S was compared, and the results are shown in Figure 6b. Figure 6b shows that the photodegradation efficiency of TC was only 2.83% without any photocatalyst, and were 55.82% and 14.94% with CdS and MnS alone, respectively, after 60 min of illumination, indicating that a single catalyst of CdS or MnS could realize the photodegradation of TC; yet the degradation efficiency of TC was very low. When MnS was doped into CdS, the photodegradation efficiency of TC reached 72.66% catalyzed by Cd_0.6_Mn_0.4_S, which was greater than that of TC catalyzed by either CdS or MnS, yet was still not satisfactory. However, after Cd_0.6_Mn_0.4_S was loaded on biochar, since biochar could adsorb TC effectively on its surface and then facilitate the reaction catalyzed by Cd_0.6_Mn_0.4_S, the photodegradation efficiency of TC catalyzed by C@Cd_0.6_Mn_0.4_S (1:1) reached 90.01%. When the mass ratio of biochar to Cd_0.6_Mn_0.4_S was 1:2 (C@Cd_0.6_Mn_0.4_S (1:2)), the degradation efficiency of TC was slightly better within 30 min, and then gradually approached that catalyzed by C@Cd_0.6_Mn_0.4_S (1:1), and was even higher, reaching 90.05%, which is 1.24 times that catalyzed by Cd_0.6_Mn_0.4_S and 1.61 times that catalyzed by CdS. The degradation efficiency data of TC catalyzed by the catalysis material was fitted using the first-order reaction kinetics model to obtain the reaction rate constant (*K*), and the results are shown in Figure 6c. Figure 6c shows that the TC degradation rate constant *K* value catalyzed by Cd_0.6_Mn_0.4_S is 0.01575 min^−1^, which is greater than that catalyzed by CdS or MnS, but it is less than the *K* value catalyzed by C@Cd_0.6_Mn_0.4_S. The order of each *K* value in terms of magnitude is as follows: *K* C@Cd_0.6_Mn_0.4_S (1:1) > *K* C@Cd_0.6_Mn_0.4_S (1:2) > *K* C@Cd_0.6_Mn_0.4_S (1:3) > *K* C@Cd_0.6_Mn_0.4_S (1:4) > *K* Cd_0.6_Mn_0.4_S > *K* CdS > *K* MnS > *K* C > *K* blank. Loading Cd_0.6_Mn_0.4_S on biochar, TC can be photocatalyzed to degrade more effectively, and as more biochar added, the *K* value increases. However, it was found in the experiment that the more biochar is present in the composite catalyst, the stronger the adsorption effect, and the recycling effect of the catalyst is relatively poor. Considering all factors, the C@Cd_0.6_Mn_0.4_S (1:2) catalyst was selected for the subsequent experiments (the photodegradation rate constant *K* value of TC catalyzed by C@Cd_0.6_Mn_0.4_S (1:2) was 0.02624 min^−1^). To illustrate the role of light, a control experiment with C@Cd_0.6_Mn_0.4_S (1:2) in the absence of light was conducted, and the result (Figure 6b) illustrates the condition of the absence of light: TC removal depends on adsorption and TC is hardly degraded. It may be that TC degradation requires light to provide the activation energy needed for the reaction.

### 3.3. Effects of Reaction Conditions on TC Photodegradation Efficiency Catalyzed by C@Cd_0.6_Mn_0.4_S

When exploring the influence of reaction conditions on TC degradation efficiency catalyzed by C@Cd_0.6_Mn_0.4_S, C@Cd_0.6_Mn_0.4_S was prepared at the theoretical mass ratio of biochar to Cd_0.6_Mn_0.4_S, i.e., 1:2. The preparation process was described in detail in Section 2.2.4.

**Figure 7:**
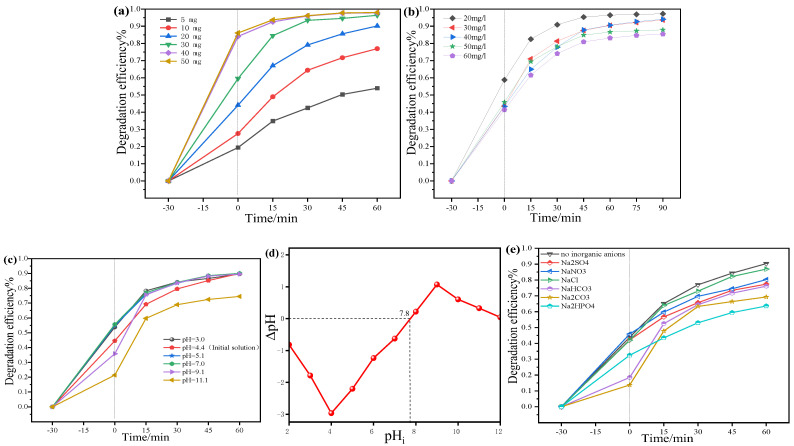
Effects of the dosage of C@Cd_0.6_Mn_0.4_S (**a**), the initial mass concentration of TC (**b**), the initial solution pH (**c**), the pHzpc of C@Cd_0.6_Mn_0.4_S (**d**), and inorganic anions and humic acids (**e**) on photocatalytic degradation of TC catalyzed by C@Cd_0.6_Mn_0.4_S (reaction conditions: C@Cd_0.6_Mn_0.4_S dosage of 20 mg, temperature of 30 °C, TC aqueous solution mass concentration of 40 mg/L and volume of 40 mL).

The influence of catalyst dosage on TC photodegradation is shown in Figure 7a. Figure 7a illustrates that when the catalyst dosage is 5, 10, 20, 30, 40, and 50 mg, and the photodegradation of 40 mL TC aqueous solution with a mass concentration of 40 mg/L lasted for 60 min under the conditions of a temperature of 30 °C and visible light illumination, the photodegradation efficiency of TC increased with the catalyst dosage increase. The corresponding degradation efficiencies are 53.95%, 76.94%, 90.17%, 96.36%, 97.79%, and 98.01%, respectively. The degradation efficiency reached the maximum when the catalyst dosage was 50 mg. However, after the catalyst dosage increased from 40 mg to 50 mg, the degradation efficiency did not increase significantly. Therefore, choosing a catalyst dosage of 40 mg is appropriate.

The influence of TC concentrations on the photocatalytic degradation efficiency of TC is presented in Figure 7b. With a dosage of C@Cd_0.6_Mn_0.4_S of 20 mg, a solution volume of 40 mL, a temperature of 30 °C, and a photocatalytic degradation time of 60 min, when the TC aqueous solution concentrations were 20, 30, 40, 50, and 60 mg/L, the degradation efficiency of TC gradually decreased with TC concentration increase, at 96.45%, 90.60%, 90.48%, 86.61%, and 83.14%, respectively. The results are due to the dosage of the catalyst remaining unchanged, and the amount of active substances produced by illumination remaining unchanged. When the volume of the TC solution to be degraded remained unchanged and the concentration increased, the number of TC molecules to be degraded increased, making the same amount of active substances insufficient to degrade all TC molecules, resulting in a decrease in the degradation efficiency. In addition, a higher mass concentration of TC may prevent the catalyst from absorbing light through shielding, further reducing the degradation efficiency. When the TC concentration was 30 and 40 mg/L, the degradation efficiencies did not differ much. Therefore, a TC concentration of 40 mg/L was chosen for the subsequent experiments.

The pH value of solutions dominates the existing forms of TC molecules and the surface charge of the catalysts, thus affecting TC degradation efficiency. Under different pH conditions, TC molecules exist in different forms (pK_1_ = 3.30, pK_2_ = 7.68, pK_3_ = 9.68). Depending on the pH value of the solution, TC molecules can be dissociated into cations, zwitter-ions, and anions. When pH < 3.30, the main form of TC is TCH_3_^+^ (cation). When 3.30 < pH < 7.68, the main form of TC is TCH_2_^0^ (ampholyte, neutral). When 7.68 < pH < 9.68, the main form of TC is TCH^-^ (anion). When pH > 9.68, the main form of TC is TC^2−^ (anion). In general, TC mainly exists in the forms of cation, ampholyte, and anion at pH = 3.3, pH = 3.3–7.8, and pH = 7.8–9.68 [38,39]. The influence of the degradation efficiency of TC catalyzed by C@Cd_0.6_Mn_0.4_S at different pH values of 3.0, 4.4 (original aqueous solution pH value), 5.1, 7.0, 9.1, and 11.1 was explored. When the initial pH of the TC aqueous solution with a volume of 40 mL and an initial concentration of 40 mg/L was adjusted to 3.0, 4.4 (original solution pH value), 5.1, 7.0, 9.1, and 11.1, respectively, by using 1 mol/L NaOH or 1 mol/L HCl solution, and C@Cd_0.6_Mn_0.4_S of 20 mg was added to catalyze photodegradation reaction of TC at 30 °C for 60 min, the degradation efficiencies of TC were 89.60%, 89.99%, 90.02%, 90.07%, 89.56%, and 74.89%, respectively (Figure 7c). When pH is between 3 and 9, the degradation efficiencies of TC do not differ much. The zero potential of C@Cd_0.6_Mn_0.4_S measured by the charge titration method is approximately 7.8 (Figure 7d). When the solution pH is less than 7.8, the surface of C@Cd_0.6_Mn_0.4_S is positively charged and substances with negative charges are more easily adsorbed on the surface of the catalyst; while when the solution pH is greater than 7.8, the surface of C@Cd_0.6_Mn_0.4_S is negatively charged, making it easier to adsorb substances with positive charges. When the solution pH is 3 to 9, TC mainly exists in the form of ampholyte, reducing the electrostatic repulsion between TC and C@Cd_0.6_Mn_0.4_S. However, when pH is 11.1, the degradation efficiency is the lowest, probably because TC mainly exists in the form of anion (TC^2–^) at a pH of 11.1, generating an electrostatic repulsion with the catalyst, thereby hindering TC from being adsorbed on the surface of the photocatalyst and ultimately leading to a decrease in the degradation efficiency. Based on the abovementioned dynamics, it can be concluded that when pH is between 3 and 9, the influence of pH on the photodegradation of TC by C@Cd_0.6_Mn_0.4_S is not significant, and there is no need to adjust the pH of TC aqueous solution.

C@Cd_0.6_Mn_0.4_ may have complex interactions with inorganic ions and organics in water environments, and whether ions and organics commonly found in natural water would influence the photocatalytic degradation of TC by C@Cd_0.6_Mn_0.4_ should be investigated. Common inorganic ions and organics, namely HPO_4_^2−^, HCO_3_^−^, NO_3_^−^, Cl^−^, CO_3_^2−^, SO_4_^2−^, and humic acid (HA), were chosen to study their effects on TC photocatalytic degradation by C@Cd_0.6_Mn_0.4_. The results are shown in Figure 7e. When 10 mmol/L of HPO_4_^2−^, CO_3_^2−^, HCO_3_^−^, SO_4_^2−^, NO_3_^−^, Cl^−^, and 10 mg/L of HA, respectively, was added to the TC solution, the corresponding degradation efficiencies were 63.61%, 69.30%, 76.12%, 77.41%, 80.25%, 86.88%, and 83.33%, respectively. The presence of NO_3_^−^, Cl^−^, and HA has little influence on the photocatalytic degradation efficiency of TC by C@Cd_0.6_Mn_0.4_, which may be because NO_3_^−^ and Cl^−^ do not shield the photocatalytic active sites. HA is a macromolecular polymer with aromatic nuclei as the main body and various functional groups attached; the main functional groups are phenolic hydroxyl groups, hydroxyl groups, methoxy groups, and cyclic compounds containing nitrogen. HA is an amphoteric colloid, both negatively and positively charged on its surface, usually mainly negatively charged, and the source of electricity is mainly the oxygen-containing group on the molecular surface, such as the dissociation of carboxyl, hydroxyl, methyl, acyl, and other groups and the protonation of the amine group. These HA’ characters may cause it to interact with TC and make HA have little influence on the photocatalytic degradation efficiency of TC. HCO_3_^−^ and SO_4_^2−^ have slight influence on the degradation TC, which is likely due to the capture of active substances by HCO_3_^−^ and SO_4_^2−^ during photocatalytic degradation [28]. The influence of CO_3_^2−^ and HPO_4_^2−^ on the degradation efficiency of TC is relatively high; possibly CO_3_^2−^ and HPO_4_^2−^ show alkalinity in the solution, causing the photocatalytic degradation efficiency of TC to be reduced, as shown in the results of Figure 7e.

### 3.4. Universality and Reusability of Photocatalytic Properties of C@Cd_0.6_Mn0_.4_S

**Figure 8:**
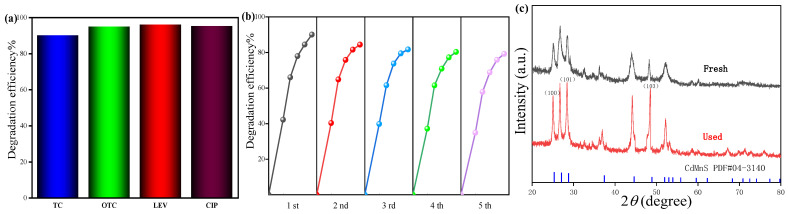
The photodegradation efficiencies of antibiotics catalyzed by C@Cd_0.6_Mn_0.4_S (**a**); cyclic stability during degrading TC (**b**), and XRD pattern before and after five cycle reactions for degrading TC (**c**). (Reaction conditions: C@Cd _0.6_Mn_0.4_S dosage of 20 mg, temperature of 30 °C, TC solution and OTC solution mass concentration of 40 mg/L, LEV solution and CIP solution of mass concentration of 20 mg/L, and the volume of each solution of 40 mL).

The photocatalytic degradation performance of C@Cd_0.6_Mn_0.4_S on the other antibiotics under the same conditions is shown in Figure 8a. As illustrated in Figure 8a, C@Cd_0.6_Mn_0.4_S has a good photocatalytic degradation efficacy not only on TC, but also on the other antibiotics such as oxytetracycline (OTC), levofloxacin (LEV), and ciprofloxacin (CIP). Their photocatalytic degradation efficiencies within 60 min are 90.21%, 95.09%, 96.19%, and 95.36%, respectively. To verify the reusability of C@Cd_0.6_Mn_0.4_S for photocatalytic degradation of TC, five cycling experiments were carried out on the photocatalytic degradation. The experimental conditions were 30 min of dark adsorption and 60 min of photocatalytic degradation. The results are shown in Figure 8b. After five cycling experiments, the photocatalytic degradation efficiency of TC by C@Cd_0.6_Mn_0.4_S gradually decreased from 90.05% to 79.23%. Comparing TC degradation efficiency by C@Cd_0.6_Mn_0.4_S with those by the reported photocatalysts, and the comparison results of TC photocatalytic degradation efficiency and the reaction conditions by C@Cd_0.6_Mn_0.4_S with those in the reported literatures by various photocatalysts, are shown in Table 1, and it is concluded that the photocatalytic degradation performance of C@Cd_0.6_Mn_0.4_S is comparable to those of the reported catalysts in the previous study in terms of the degradation efficiency of TC. In addition, the main reason for the decrease in photocatalytic efficiency observed in the reusability test is that the catalyst C@Cd_0.6_Mn_0.4_S experiences a slight mass loss in the process of recycling, and TC or its degradation intermediates would occupy little of the active sites of the catalyst. The XRD pattern of C@Cd_0.6_Mn_0.4_S before and after photocatalytic degradation of TC is shown in Figure 8c. Compared with the catalyst before the reaction, the main peaks of the catalyst after five reactions exhibit almost no change, except that XRD peaks of the used C@Cd_0.6_Mn_0.4_S sample narrowed, indicating that the crystal phase of C@Cd_0.6_Mn_0.4_S remained intact after the reaction, confirming that C@Cd_0.6_Mn_0.4_S has good stability. XRD peaks of the used C@Cd_0.6_Mn_0.4_S sample narrowed, which is supposedly evidence of recrystallization, which took place after the sample dried for 12 h at 70 °C.

### 3.5. Photocatalytic Degradation Mechanism

**Figure 9:**
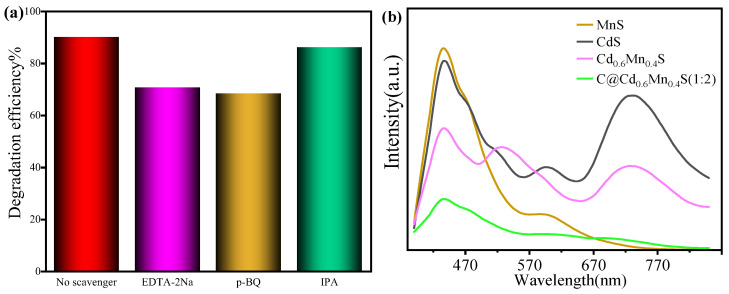
Effects of different quenchers on degradation efficiencies of TC (**a**) (Reaction conditions: C@Cd_0.6_Mn_0.4_S dosage of 20 mg, TC solution mass concentration of 40 mg/L, solution volume of 40 mL and temperature of 30 °C), and fluorescence intensity of the prepared material (**b**).

In the C@Cd_0.6_Mn_0.4_S photocatalytic degradation of the TC system, 2 mmol/L disodium ethylenediaminetetraacetate (EDTA-2Na), *p*-quinone (*p*-BQ), and isopropyl alcohol (IPA) were added as the scavengers for photogenerated holes (h^+^), superoxide radicals (•O_2_^−^), and hydroxyl radicals (•OH), respectively. The influence of the scavengers on TC degradation efficiencies was analyzed. The results are shown in Figure 9a. EDTA-2Na (h^+^ scavenger) and *p*-BQ (•O_2_^−^ scavenger) have inhibitory effects on the degradation of TC, indicating that in the reaction system, both •O_2_^−^ and h^+^ are active substances of the reaction and directly participate in the degradation of TC molecules. However, the addition of IPA (•OH scavenger) has little influence on TC degradation efficiency, indicating that in this reaction system, •OH contributes little to the degradation of TC.

**Scheme 2:**
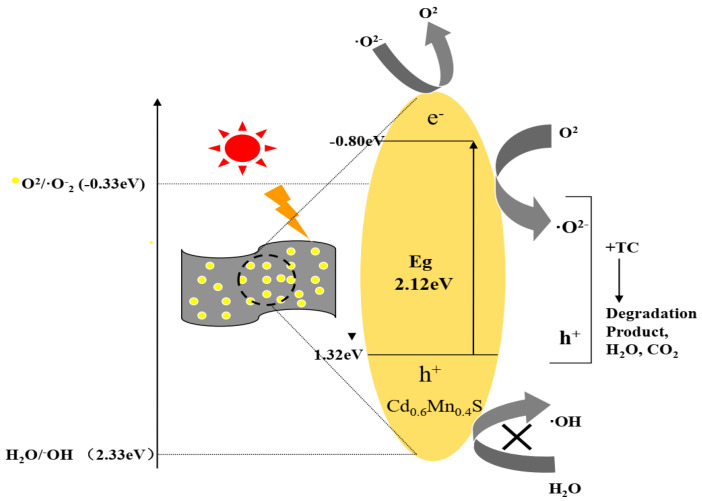
Schematic diagram of the photocatalytic degradation mechanism of TC by C@Cd_0.6_Mn_0.4_S under visible light irradiation.

The photoluminescence (fluorescence) intensity can reflect the recombination degree of charge carriers produced by the material exposed to light. The higher the intensity of photoluminescence, the higher the recombination rate of electrons and holes, and the lower the photocatalytic performance. Figure 9b illustrated that the luminescence spectrum shape of C@Cd_0.6_Mn_0.4_S is almost the same as that of the Cd_0.6_Mn_0.4_S, and the luminescence intensity of C@Cd_0.6_Mn_0.4_S and Cd_0.6_Mn_0.4_S is lower than that of the CdS monomer. The order of luminescence intensity from strong to weak is: CdS > Cd_0.6_Mn_0.4_S > C@Cd_0.6_Mn_0.4_S, and there is no fluorescence on the biochar. The results indicate that Mn^2+^ doped and biochar support strategies can effectively inhibit the recombination rate of photoexcited electrons and holes, which is consistent with the changing trend of the photocatalytic activity of the obtained composite materials. These results may be due to the loose and porous structure of biochar from biomass gasification carbon residue, which makes it easier for Cd^2+^ and Mn^2+^ to disperse and anchor on the surface of biochar. Furthermore, biochar has excellent electrical conductivity; the two factors significantly increase the binding rate of Cd^2+^ and Mn^2+^, the dispersity of Cd_0.6_Mn_0.4_S, and the charge transfer of the material, thereby enhancing the photocatalytic performance of Cd_0.6_Mn_0.4_S. Based on the above analysis, the possible mechanism of photocatalytic degradation of TC by C@Cd_0.6_Mn_0.4_S is speculated as shown in Scheme 2.

Under the irradiation of visible light whose energy is greater than or equal to the bandgap energy, C@Cd_0.6_Mn_0.4_S can generate photo-generated electrons (e^−^) and holes (h^+^), and electrons (e^−^) in the valence band (VB) will be excited and transited to the conduction band (CB). At the same time, holes (h^+^) are left on the VB as active species. Since Cd_0._6Mn_0.4_S is loaded on biochar, more active sites are provided, which increases the contact chance between Cd_0.6_Mn_0.4_S and TC, and increases the exposure of the active sites of Cd_0.6_Mn_0.4_S, realizing the effective separation of electrons and holes, hindering the rapid recombination of photogenerated electrons and holes, and thereby improving the photocatalytic activity. Since the CB potential of Cd_0.6_Mn_0.4_S is more negative than that of E (O_2_/•O_2_^−^ = −0.33 eV) [37,46,47], the electrons (e^−^) on CB of Cd_0.6_Mn_0.4_S will quickly migrate to the surface of biochar and react with O_2_ adsorbed on the active site to form •O_2_^−^, and then react with TC molecules to decompose into small molecules. With the continuous transfer of electrons, holes (h^+^) are left on VB of Cd_0.6_Mn_0.4_S. These remaining h^+^, due to their strong oxidation ability, will directly participate in the oxidation decomposing process of TC and play a major role in photocatalytic degradation. Eventually, TC is oxidized, degraded into small molecules, substances, and even into CO_2_ and H_2_O, etc. The mechanism is shown in Formulas (3)–(5).

C@Cd_0.6_Mn_0.4_S + h*v* → C@Cd_0.6_Mn_0.4_S(e^−^(CB) + h^+^(VB)) (3)

e^−^ + O_2_ → •O_2_^−^ (4)

TC + (•O_2_^−^ + h^+^) → small organic molecules → CO_2_ + H_2_O. (5)

## 4. Conclusions

1. The C@Cd_x_Mn_1−x_S composite catalyst was prepared by hydrothermal method using biomass gasification residues, Cd (OAc)_2_·2H_2_O, Mn (OAc)_2_·4H_2_O, and TAA as raw materials.
2. The Cd_x_Mn_1−x_S prepared with a mole ratio of Cd to Mn at 6:4 has the good degradation efficacy for TC in response to light reaction. After 60 min of visible light irradiation, the degradation efficiency of TC reaches 72.66%, but it is still not ideal. The C@Cd_0.6_Mn_0.4_S prepared after Cd_0.6_Mn_0.4_S loaded on biochar has the highest degradation efficiency of 90.05% after light irradiation of 60 min when the mass ratio m(biochar):m(Cd_0.6_Mn_0.4_S) is 1:2. After being recycled 5 times, the degradation efficiency of TC reaches 79.23%, which is still higher than the catalytic effect of the catalyst Cd_0.6_Mn_0.4_S. The composite catalyst has good cycling stability.
3. C@Cd_0.6_Mn_0.4_S is applicable to a wide pH environment (3–9), and NO_3_^−^, Cl^−^, and HA had little effect; HCO_3_^−^ and SO_4_^2−^ had a slight effect; and CO_3_^2−^ and HPO_4_^2−^ had a relative high effect on the degradation of TC.
4. Both •O_2_^−^ and h^+^ directly participate in the photodegradation of TC molecules, and •OH contributes little to the photo degradation of TC catalyzed by C@Cd_0.6_Mn_0.4_S.
5. C@Cd_0.6_Mn_0.4_S has universality for different antibiotics. The degradation efficiencies of 20 mg/L levofloxacin, ciprofloxacin, and 40 mg/L oxytetracycline catalyzed by C@Cd_0.6_Mn_0.4_S ranged from 89.88% to 98.69%.
6. Biomass gasification residues, as the carrier of the catalyst, can significantly enhance the dispersibility of Cd_0.6_Mn_0.4_S nanoparticles, expose more reaction sites of the catalyst, and improve the photocatalytic activity.

## Figures and Tables

**Table 1:** Comparison of TC photocatalytic degradation efficiency by C@Cd_0.6_Mn_0.4_S with those by the reported photocatalysts.

Catalyst	TC	Reaction Conditions	Degradation Efficiency	Degradation Efficiency After Cycles	Ref.
Bi_12_O_17_Cl_2_	0.02 g/L,40 mL	catalyst = 0.025 g/L	60 min, 92.2%	60 min, 80.5%	[40]
Zn-HT-CN	0.02 g/L,10 mL	catalyst = 0.3g/L	40 min, 94.41%	40 min, 78.78%	[41]
In_2_O_3_Nanoparticl/Bi_4_O_5_Br_2_	0.035 g/L,100 mL	catalyst = 0.02 g/L	100 min, 92.4%	100 min, 82.4%	[42]
Zn_0.2_Ti_0.8_-NML	0.02g/L,100 mL	catalyst = 0.02 g/L	40 min, 95.6%	40 min, 85%	[43]
MCN@CdS	0.02 g/L,100 mL	catalyst = 0.02 g/L in presence of O_2_	80 min, 99.5%	80 min, 90%	[44]
Ce−Bi_2_WO_6_	0.001 g/L,50 mL	catalyst = 0.6 g/L, pH = 6.7	40 min, 90.4%	40 min, 89%	[45]
C@Cd_0.6_Mn_0.4_S	0.04 g/L40 mL	catalyst = 0.02 g/L, pH = 5.1	60 min, 90.05%	60 min, 79.23%	This work

## Data Availability

The original contributions presented in the study are included in the article, further inquiries can be directed to the corresponding author.
